# The Potential Impact of Probiotics on the Gut Microbiome of Athletes

**DOI:** 10.3390/nu11102270

**Published:** 2019-09-21

**Authors:** Laura Wosinska, Paul D. Cotter, Orla O’Sullivan, Caitriona Guinane

**Affiliations:** 1 Department of Biological Sciences, Cork Institute of Technology, Bishopstown, T12 P928 Cork, Ireland; Laura.Wosinska@teagasc.ie; 2 Teagasc Food Research Centre, Moorepark, Fermoy, P61 C996 Cork, Ireland; paul.cotter@teagasc.ie; 3 APC Microbiome Ireland, T12 YT20 Cork, Ireland

**Keywords:** athletes, probiotic, microbiome, overtraining, fitness, exercise

## Abstract

There is accumulating evidence that physical fitness influences the gut microbiome and as a result, promotes health. Indeed, exercise-induced alterations in the gut microbiome can influence health parameters crucial to athletic performance, specifically, immune function, lower susceptibility to infection, inflammatory response and tissue repair. Consequently, maintenance of a healthy gut microbiome is essential for an athlete’s health, training and performance. This review explores the effect of exercise on the microbiome while also investigating the effect of probiotics on various potential consequences associated with over-training in athletes, as well as their associated health benefits.

## 1. A Brief Introduction to Exercise and the Microbiome

Our gut microbiome, an elusive and recently designated organ, is under ever greater scrutiny. Uncovering the significance of the microbiome in human health and disease has become possible with the advent of various genomic tools accompanied by improvements in culture-based approaches (culture-omics). The gut contains a rich and diverse microbial ecosystem whose activities can influence the health of the host [1]. More specifically the gut is home to trillions of microbial species [2] that co-exist with human cells. The dominant microbial populations in the gut are bacteria from the Bacteroidetes and Firmicutes phyla, followed by members of the phyla Actinobacteria, Proteobacteria and Verrucomicrobia. The microbiome also consists of a fungal community, including species of *Candida* and *Saccharomyces* [3], viruses (primarily bacteriophage) [4], as well as members from the archaeal domain [2]. Despite the microbiome being stable in adults, certain factors can alter its structure, including, diet, antibiotics, probiotics and indeed exercise/fitness (for review see [5,6]). The microbiome appears to have an effect on most aspects of human health. In addition to a role for specific microbes, or groups of microbes, the importance of microbial diversity has been recognized in many studies, with a decreased diversity associated with a number of gastrointestinal (GIT) conditions such as Crohn’s disease [7], certain cancers [8] and Type 1 diabetes [9]. An altered microbiome can affect energy metabolism, immune function and oxidative stress, all of which are vital for athlete’s performance and overall health (for review see [10]). A potential, non-medical, route to maintaining athlete’s health is through probiotics. Probiotics are defined as live bacteria that, when ingested in adequate amounts, confer a benefit to the host [11]. Although such health benefits have frequently been credited to specific bacterial strains from the genera *Bifidobacterium and Lactobacillus,* the potential of next-generation probiotic (NGP) candidates and designer probiotics is also being recognized.

Although it is generally accepted that microbial diversity is an indicator of human health, this hypothesis has been challenged recently by Shade, who postulated that “mechanisms maintaining or changing microbial diversity are many and complex” and assumptions that an increased diversity is better may oversimplify complex mechanisms of health and disease [12]. As previously stated, certain factors including an individual’s lifestyle can alter the structure of the microbiome. It has been noted across a number of studies that athletes have increased gut microbial diversity compared with more sedentary controls [13,14,15]. Clarke et al. [14] demonstrated significant gut microbiome differences between male professional elite rugby players and a cohort of non-athlete male controls. Notably, the athletes had significantly higher gut microbial diversity accompanied by elevated levels of the *Akkermansia* genus. A subsequent study of the same cohort [13] concluded that athletes had a higher abundance of short-chain fatty acids (SCFA) metabolic pathways. In another instance, Petersen et al. [15], investigated the gut microbiome of cyclists and concluded that 30 out of 33 cyclists also had an increased abundance of *Akkermansia*. It was also observed that exercise regime is proportional to the abundance of *Prevotella*, which in turn has been associated with increased branched-chain amino acid (BCAA) pathways, important in muscle recovery. There was also an increase in *Methanobrevibacter smithii* within the professional cyclist group compared to amateur cyclists, a microbe that utilises H_2_ in the colon to make SCFA and Adenosine triphosphate (ATP) [15]. More recently, Cronin et al. investigated the impact of exercise and/or whey protein supplementation over an eight-week period on a cohort of sedentary adults. In this instance, the differences between the microbial communities of the three test populations were more subtle, a fact that could be attributed to the shorter period over which time these interventions took place relative to the potentially life-long practices of some athletes, i.e., microbial architecture is not easily changed and it may take more time to notice significant impacts [16]. Recently, Jang et al. compared the gut microbiome of bodybuilders, distance runners and controls (*n* = 45), each of the groups were ingesting a different sport specific diet. The results show that bodybuilders ingesting a high-protein and fat diet had increased relative abundance of *Faecalibacterium*, *Sutterella*, *Clostridium*, *Haemophilus* and *Eisenbergiella*, but decreased abundances of *Bifidobacteria*, *Parasutterella* and *Eubacterium*. The study also concluded that individuals practicing anaerobic sports show similar gut microbiome patterns to bodybuilders [17]. A pioneering study by Scheiman et al. discovered that the genus *Veillonella* was enriched in the gut microbiome of marathon runners, compared to non-runners. Furthermore, using a mouse model they demonstrated that *Veillonella atypica* increased endurance, reduced inflammatory cytokines, and converted lactate to acetate/propionate [18]. The present studies suggest that exercise can alter the microbiome in a beneficial manner, where the alteration in the composition of the microbiome can be associated with increased health parameters and thus possibly affect athletic performance in a beneficial way. Reported gut microbiome changes/differences associated with exercise and fitness are summarized in Table 1.

Whilst exercise confers numerous physiological effects on the host including mood regulation [19], improving cardiovascular symptoms [20], alleviation of fatigue [21] and anti-inflammatory effects, a healthy balance between training load and recovery needs to be maintained to prevent the condition of overtraining occurring [22]. Studies in the past have observed the effect of overtraining on the microbiome; Allen et al. concluded that voluntary and forced exercise altered the mice microbiome in different ways. Of particular interest were the phyla *Tenericutes* and *Proteobacteria,* both elevated in forced exercised mice, compared to mice that exercised voluntarily. *Tenericutes* spp. have been linked to intestinal inflammation in human subjects and *Proteobacteria* bacteria are known for their lipopolysaccharide production [23]. Similarly, Karl et al. observed that there was a large increase in pathogenic taxa post-exercise in soldiers including, *Peptostreptococcus, Staphylococcus, Peptoniphilus, Acidaminococcus*, and *Fusobacterium* and a decrease in more beneficial taxa *Bacteroides, Faecalibacterium, Collinsellaa* and *Roseburia*. The authors postulated that the increase in more toxic species may possibly explain the observation of increased intestinal permeability within the soldier cohort [24]. More recently, Yuan et al. concluded that excessive exercise had a negative impact on microbial diversity in murine models [25].

**Figure 1:**
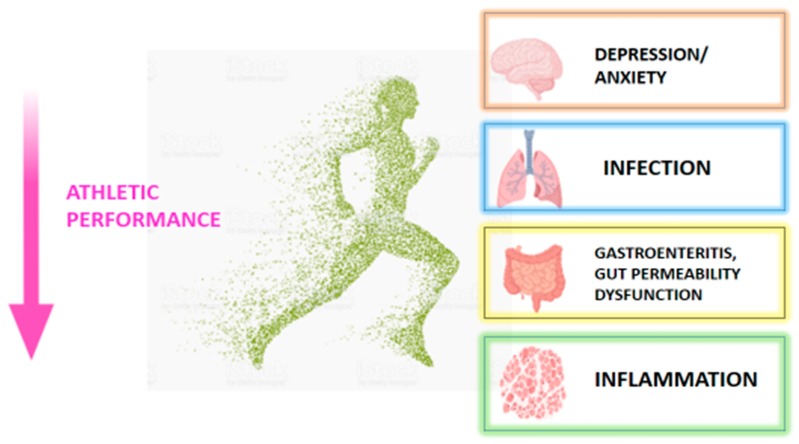
The effects of overtraining on the wellbeing of an athlete.

Various studies throughout the years have investigated overtraining and its effect on human health particularly host immunity [26,27] and susceptibility to infection [22,28,29]. The potential side effects of overtraining are illustrated in Figure 1. Furthermore, overtraining has been associated with an increased incidence of mental illnesses in athletes [30,31,32,33] and oxidative stress [34,35], where studies in animal models have shown the relationship between the microbiome and oxidative stress [36,37]. One of the most common side effects of overtraining is gut dysfunction and it has been frequently noted in athletes [38,39]. Gastrointestinal (GIT) symptoms are frequently observed with athletes who tend to travel for training or competition, purposes [40,41]. Antibiotics can be used to overcome the GIT infections, however, certain complications may arise when using antibiotics. Firstly, studies have shown that the microbiome takes about six weeks to resemble its original state post antibiotic therapy [42], it increases possible tendon rupture [43] and can cause antibiotic associated diarrhea [44].

The review aims to provide a panoramic view of what is currently known about probiotics and their effect on gut microbiome of athletes. A recent systematic review by Moller et al. [45] has provided an overview of the effects of traditional probiotics on physically active individuals and athletes, with a particular focus on the clinical trials performed to date. Here we explore the relationship between fitness and the gut microbiome focusing particularly on overtraining. We also explore the available literature on how, possibly, probiotics can help to alleviate or prevent the different side effects of overtraining. We also discuss next generation and designer probiotics, as well as the safety and toxicity considerations associated with novel strains.

## 2. Traditional Probiotics

Traditionally, the majority of probiotic strains were representatives of the lactic acid bacteria group (LAB) i.e., *Bifidobacterium, Lactobacillus* [46], however other bacterial and yeast strains are also commonly used such as *Escherichia coli* and *Saccharomyces* respectively. Although probiotics have existed for quite some time, it is only recently that their commercial potential has been realized, with the market expected to exceed $67 billion by 2024 [47]. Probiotics can consist of one or a combination of a few strains, either as capsules, powders or as a component of a food e.g., yoghurt [48]. One of the main concerns relating to the probiotic market is that some strains do not have substantial proof of efficacy. It is also important for consumers to be aware that the benefits of one probiotic strain cannot be inferred to another [48,49]. Nonetheless, there is increasing evidence of the ability of specific probiotics to, for example, modulate the immune system, impact on tight-junction proteins and inhibit pathogen colonization; this list is not exhaustive, and the benefits of probiotics are strain-dependent. Focusing on athletes as noted above, extreme exercise can be associated with undesirable symptoms and specific probiotics can help to ease or prevent certain gastrointestinal disorders, help with the brief immunosuppression period, and reduce susceptibility to infections. Ways in which existing and novel probiotics could contribute to athlete health are provided in Table 2.

### 2.1. Escherichia Coli

*Escherichia coli*, is the most diversely studied prokaryotic model organism in science. *E. coli* belongs to the *Enterobacteriaceae,* a large family of Gram-negative bacteria consisting of commensal and pathogenic bacteria, *E. coli* is a facultative anaerobe, meaning it can either respire or ferment, depending on the environment, which maximises its growth in the gut. *E. coli* Nissle 1917 (EcN) possesses antagonistic properties against *Salmonella, Yersinia, Shigella and Listeria* [50]. A study by Wehkamp found that EcN induces the production of defensin in epithelial cells [51]. Various studies have also demonstrated that *E. coli* affects the intestinal epithelial barrier [52,53], additionally EcN can be used for irritable bowel disorder [53], constipation (for review see [54]), and has pro-inflammatory potential [55]. EcN has been shown to repair the gut barrier function in vitro [53] and in murine models [52]. Henker et al, investigated whether EcN is an effective therapeutic agent against acute diarrhea in infants and toddlers. The randomized double blind placebo controlled trial, including 113 subjects concluded that, EcN successfully reduced the duration and incidence of diarrhea compared to placebo, and improved the general health of the subject [56].

EcN has shown great promise in mitigating effects of gut barrier dysfunction, diarrhea and impairment immune system, experienced often with the condition of overtraining. In order to prove its efficacy more research is needed, especially involving athlete cohorts.

### 2.2. Lactobacillus

*Lactobacillus* species are Gram-positive bacteria belonging to the lactic acid bacteria group, capable of lactic acid fermentation metabolism. *Lactobacilli* are frequently resistant to bile salts; an important probiotic trait as it allows for the probiotic to survive in the hostile acidic environment of the gastrointestinal tract [57]. *Lactobacilli* are frequently found in various fermented foodstuffs, silage, human gut and the vagina [49,50,51,52,53,54,55,56,57,58]. *Lactobacilli* are amongst the most widely used and characterized probiotics to date. Various strains from the species: *Lactobacillus rhamnosus, Lactobacillus plantarum* and *Lactobacillus acidophillus* have been considered important probiotics, each strain exhibiting individual functions. A small selection of very many examples are provided here, *Lactobacillus casei* GG has been found to shorten diarrheal distress in subjects suffering from viral gastroenteritis [59], *Lactobacillus johnsonii* BFE 6128 and *Lb. plantarum* BFE 1685 have been shown to aid in modulating the immune system by inducing the secretion of the cytokine IL-8 in vitro [60], *Lb. rhamnosus* 4B15 and *Lactobacillus gasseri* 4M13 have been suggested to inhibit the expression of inflammatory cytokines at transcriptional level in vitro thus showing anti-inflammatory potential [61]. Among the aforementioned benefits, *lactobacilli* are capable of inhibiting enteric pathogens through the production of lactic acid, bacteriocins and hydrogen peroxide [62]. It is worth to mention that *lactobacilli* are biofilm matrix formers, which makes the bacteria more resistant to antibiotics, and could potentially allow for longevity of the bacteria in the gut [63].

Most studies to date have investigated the potential impact of *lactobacilli* in either animal models or non-active human populations and, as of today, little has been done in athletes. Cox et al investigated whether *Lb. fermentum* VRI-003 had any effect on mucosal immunity in elite male distance runners. They observed a significant decrease in the duration and severity of respiratory illness, and a two-fold increase in interferon gamma (INFƴ) [64]. Another study examined *Lb. fermentum* (PCC^®^) and its effects on gastrointestinal and respiratory health in competitive cyclists. In this study West and colleagues deduced that *Lb. fermentum* (PCC^®^) was successful in reducing the severity of gastrointestinal symptoms in male cyclists, in addition, it significantly reduced (20%–60%) cytokine imbalance caused by acute exercise. Moreover, the study also concluded that the severity and duration of lower respiratory illness decreased in male cyclists but increased in female cyclists, taking the probiotic compared to placebo [65]. It is also worth noting that a recent study by Shing et al, reported that a multi-strain probiotic formulation consisting of *Lb. acidophilus, Lb. rhamnosus, Lb. casei, Lb. plantarum, Lb. fermentum, B. lactis, B. breve, B. bifidum and Streptococcus thermophilus* increased run to fatigue time in male runners, and showed small to moderate improvement in gut permeability [66]. Overtraining can result in a compromised immune system and gut barrier function; in particular *lactobacilli* are capable of reducing the severity and duration of upper respiratory tract infections (URTI) and gastrointestinal problems, as well as modulating the immune system and increasing performance and thus a good candidate probiotic for athletes.

### 2.3. Bifidobacterium

*Bifidobacteria* are Gram-positive anaerobic bacteria from the phylum Actinobacteria. Associated strains are typically among the first colonisers of the infant gut and contribute to the infants’ immune system maturation and the utilisation of certain dietary components [67]. They are generally bile-acid resistant [68] and represent a large proportion of the bacterial microbiome obtained from infant faecal samples, on average about 60%–91% present in breast fed infants, but proportions decrease greatly later in life [69]. Numerous strains from the species: *Bifidobacterium breve, Bifidobacterium longum, Bifidobacterium bifidum* exhibit probiotic properties, several examples are listed below. The *Bifidobacteria* genus is represented by many different strains that have been assigned probiotic properties, shown here is a small selection of strains, although many more have been noted in literature A mixture of *B. breve, B. longum and Bifidobacterium infantis* has been associated with the treatment of antibiotic associated diarrhea [70], while two *Bifidobacterium* strains: *B. breve* strain Yakult and *B. bifidum* strain Yakult have been shown by in vitro studies to have a possible role in modulating the immune system [71]. Even though *bifidobacteria* are frequently used in probiotic preparations and many studies have elucidated their benefits in vivo, little has been studied in athlete populations. West et al investigated whether a single strain of a probiotic (*B. animlais* subsp. *lactis* BI-04) compared against a multi-strain probiotic (*Lb. acidophilus* NCFM and *B. animalis* subsp. *lactis* BI-04) was effective in reducing the risk of upper respiratory tract infection in physically active individuals. The study concluded that *B. animalis* subsp. *lactis* BI-04 reduced the risk of upper respiratory illness by 27%. The multi-strain probiotic did not show a significant decrease in risk of URTI [72]. Another worthwhile study investigated a multi-strain probiotic composed of *B. bifidum*, *B. longum* and *Lb. gasseri* and its effect on the duration and incidence of infection in elite rugby players. The findings were that the multi-strain probiotic formulation reduced both the incidence and duration of URTI and gastrointestinal symptoms [73]. In addition to the aforementioned studies, Jäger and colleagues concluded that a probiotic composed of *B. breve* BR03 and *S. thermophilus* FP4 has a positive effect on the reduced performance and range of motion followed by intense muscle damaging exercise [74]. More recently, Pugh et al. demonstrated that a four week supplementation of *Lb. acidophilus* (CUL 60/ CUL 21), *B. bifidum* (CUL20) and *B. animalis* subsp. *Lactis* (CUL34) successfully lowered the incidence and severity of GIT symptoms both during training and a marathon race [75]. *Bifidobacteria* supplementation can positively help with the burden of gastrointestinal distress found often in overtrained athletes.

### 2.4. Saccharomyces

*Saccharomyces* is another commonly used probiotic; it is a non-pathogenic yeast, and is one of the most common fungal species found in the microbiome [3]. The gut mycobiome represents about 0.1% of the total microbiome [76]. *Saccharomyces boulardii* is most widely used for the treatment of traveler’s diarrhea [77]; it can prevent antibiotic-associated diarrhoea and treat recurrent *Clostridium difficile* infections [78]. Additionally, research suggests that taking *S. boulardii* benefits irritable bowel syndrome sufferers [79]. It is not to be overlooked that immunocompromised patients are at risk of fungemia, if supplementing with *Saccharomyces* [78]. The effect of *S. boulardii* on diarhoea was elucidated by Kurugol and Koturoglu, where they enrolled 200 young children in a randomized placebo study. The study concluded that *S. boulardii* successfully reduced the duration of diarrhea and hospital stay associated with it, compared to placebo [80]. In a similar study, Billoo et al. investigated *S. boulardii* and its possible impact on diarrheal episodes in 100 children, the study concluded that the probiotic reduced the number of diarrhea episodes by 50%, lowered stool frequency and the duration of the illness [81].

It can be postulated that *S. boulardii* could possibly be an effective treatment for athletes and their reoccurring gastrointestinal problems.

**Table 2:** Traditional probiotics and next-generation probiotics and their benefits.

Probiotic Genus *	Found in the Body	Dietary Source:	Potential Benefits Attributed to Specific Strains	References
*Lactobacillus*	Colon, gut and vagina	Yoghurt, fermented foods, bread, sauerkraut, wine etc.	Gastroenteritis, easing lactose intolerance, immune system modulation, alleviating inflammation, lowering cholesterol, cancer protection, modulating brain activity, preventing pathogen colonisation, bile resistant.	[60,61,62,68,82,83,84,85,86,87]
*Bifidobacterium*	Colon, oral cavity, breast milk and vagina	Yoghurt, kombucha, sauerkraut, kefir etc.	Bile resistant, easing lactose intolerance, antibiotic-associated diarrhoea, eczema, immune system modulation, cholesterol lowering abilities	[68,71,88,89,90]
*Saccharomyces*	Colon, decaying fruit, plants, soil, insects	Wine, yoghurt, kombucha, sauerkraut etc.	Travellers’ diarrhoea, antibiotic-associated diarrhoea, preventing recurring *Clostridium difficile* infections, irritable bowel syndrome	[77,78,79]
*Escherichia coli*	Colon	Capsules	Antagonistic properties against a variety of pathogens, production of defensin, tight-junction protein modification, irritable bowel disorder, constipation, pro-inflammatory properties and colon cancer	[50,51,52,53,54,91]
*Bacteroidetes*	Colon	-	Immune system modulation, intestinal homeostasis	[92,93,94]
*Akkermansia*	Colon	-	Gut barrier function, fat mass storage, glucose homeostasis, immune system stimulation, production of Vitamin B12	[95,96,97,98,99,100,101]
*Faecalibacterium*	Colon	-	Immune system modulation, ease inflammation	[102,103]
*Eubacterium*	Colon	-	Improve insulin sensitivity, increase energy production, produce Vitamin B12, maintain intestinal homoestasis, colon detoxification, reducing the symptoms of colitis	[104,105,106,107]

* Some of the mentioned genera are regarded as potential probiotics.

## 3. Next-Generation Probiotics

Next-generation probiotics still merit the definition of a probiotic and although they have not been used as health modulators to date, have potential to be of importance to the probiotic market. Next-generation probiotics are outside of the commonly used probiotic spectrum (*Lactobacilli, Bifidobacteria* etc.), however large-scale genomic initiatives have identified putative probiotic strains with potential health benefit, mainly from the genera *Bacteroides, Akkermasia*, *Faecalibacterium* and *Eubacterium.*

One promising candidate is a strain of *Bacteroides fragilis* which has been proposed to stimulate T-cell immune responses in vitro [108], this is of interest as athletes have decreased numbers of T cells following intensive anaerobic exercise [109]. Similarly, strains of, *Bacteroides acidifaciens* have been suggested to induce IgA production in murine models and as a consequence elevating the production of IgA+ B cells and B cells; this is important because IgA plays a pivotal role in upholding intestinal homeostasis namely preventing the adherence of pathogens in the intestine [94], studies in the past have demonstrated the decrease in salivary IgA in endurance athletes [110,111] as well as when decreased it has been linked to a higher incidence of URTI [112]. Studies have illustrated the potential role of *Akkermanisa* in obesity, diabetes and inflammation (for review see [96]). *Akkermansia muciniphilia* has been associated with maintaining gut barrier function, which can be compromised in endurance athletes [113,114], and glucose homeostasis [97,98] and its direct correlation to athletic performance [115] and additionally it has the potential to stimulate the immune system [97,99,100,101]. The most recent study has concluded that *A. muciniphilia* is capable of synthesising Vitamin B12 de novo, however whether humans can benefit from the product remains unclear [95]. Considering that recent studies demonstrated an increase in *Akkermansia* in athletes this is a species that definitely warrants further investigation for use in athletes [13,14,15]. Another potential probiotic is the *Faecalibacterium prausnitzii*. *F. prausnitzii* A2-165 has been proposed to have immunodulatory capabilities, through induction of IL-10 and T-cell responses in human and murine dendric cells [102,103,116]. Another worthy strain belongs to the genus *Eubacterium. Eubacterium hallii* L2-7 has been demonstrated to improve insulin sensitivity and increase energy metabolism in obese and diabetic murine models [104], *E. hallii* DMS 3353 and *E. hallii* DSM 17630 have been indicated to produce Vitamin B12 and maintain intestinal homeostasis through the utilisation of glucose and various fermentation intermediates like acetate and lactate in vitro studies [105], while, *Eubacterium limosum* JCM 6421 has been suggested to produce SCFAs that have been shown to decrease the levels of the pro-inflammatory cytokine IL-6 and increase mucosal integrity [107].

Preliminary research has shown the benefit of the various new generation probiotics however more proof is required to ensure their efficacy, as well as testing for their safety in humans. The potential benefits of next-generation probiotics are also summarized in Table 2.

## 4. Designer Probiotics

In recent years, research has uncovered numerous functions provided by probiotics, including antagonistic activities, anti-inflammatory and tight-junction modification all of which are of particular benefit to athlete health. Designer probiotics are simply commensal strains of bacteria that have been engineered or modified in a way that they are able to resist the countless stresses that they meet both outside and inside our bodies (lyophilisation/manufacturing/acids/temperature) or simply just to improve the functions most beneficial to the host. The advent of synthetic biology has allowed for engineering of both probiotic strains and commensal strains to acquire and execute new functions (for review see [117]). The most recent concept in the probiotic field is the idea of a “biodrug”, which essentially allows for oral administration of a live recombinant probiotic strain of bacteria for the treatment/prevention of diseases. In the past 10 years, recombinant probiotics have been engineered, for the delivery of therapeutic molecules (usually proteins, fragile in nature, easily denatured by changes in the environment) such as DNA, peptides, single-chain variable fragments, various enzymes or cytokines. Probiotics are excellent vectors of transmission for such therapeutics, due to; a) their ease of colonization and direct delivery to the mucosa; b) resistance to gastric acid and bile salts; c) continued colonization and longevity of protection against pathogens; d) cost of delivery being relatively non-expensive; e) prolonged shelf-life and stability (for review see [118]). Designer probiotics could potentially be an attractive proposition to athletes due to their amenability to being tailored to the athletes specific needs.

*Lb. plantarum* NC8 which has been engineered to possess angiotensin-converting enzyme inhibitory peptides (ACEIPs), showed reduction of high blood pressure in rats [117]. It has also been shown that engineering *Lactococcus lactis* NZ29000, *B. longum* and *Lb. gasseri* ATCC 33323, managed the symptoms of diabetes and induced insulin secretion. More probiotics are currently under investigation for pathogen infection, cancer or human immunodeficiency virus (HIV), however certain issues arise with engineering probiotics [117]. Tackling the problem of biocontainment should be addressed to prevent the spread of genetically modified microorganisms (GMO) organisms into the natural environment [118] and, of course, the consumer acceptance of GMO products is still a major issue. With that being said, designer probiotics are a great alternative to todays, often impermanent medication.

## 5. Safety Considerations

When a novel probiotic strain is being considered, evidence needs to be presented to prove its efficacy and safety for human consumption. Even though probiotics are generally considered safe for use, certain side effects may arise. In a statement released by the World Health Organisation (WHO) and the Food and Agriculture Organisation of United Nations (FAO) in 2002, probiotics in theory can have four possible side effects: 1) they can be responsible for lateral gene transfer of antibiotic resistant genes i.e. *Lactobacillus* spp. 2) They can be responsible for systematic infections i.e. Fungemia. 3) They can stimulate the immune system excessively due to their immune system modulation potential, possibly causing inflammation; and 4) they are detrimental to metabolic activities, as some strains produce D-lactate which can be linked to d-acidosis (for review see [119]). However, with the advent of whole genome sequencing some of these issues can be identified or at least indicated using bioinformatics tools. Genus, species and strain level need to be provided, the nomenclature must agree with the International Committee of Systemics of Prokaryotes, and covered by the International Code of Nomenclature (ICN) of Prokaryotes for bacteria, for fungi, the nomenclature is covered by the ICN for algae, fungi and plants, the organism under consideration should be deposited in a recognized culture collection and identified by the means of current methodologies such as whole-genome sequencing (WGS). As well as using WGS to identify the organism, sequencing should be used to fully analyse the genetic content of the probiotic (both chromosomal and extra-chromosomal) to determine the presence of any transferable antimicrobial resistance loci, virulence factors or production of adverse metabolites that might deem the strain unsafe for human consumption [120]. The European Food Safety Authority (EFSA) guidelines also require full antimicrobial resistance (AMR) testing. AMR testing requires two sets of data, firstly the phenotypic testing determining the minimum inhibitory concentration (MIC) of the antimicrobial, using internationally recognized standards (ISO or similar), serial two-fold dilutions should be applied using relevant controls. The results should be compared to established and published MIC cut off values. Secondly, WGS is required to test for the presence of known AMR genes. Probiotic supplements are sold in many different doses depending on the product and strain. Most commercially available probiotics contain about 1–10 billion colony forming units (CFU) per serving. Generally, the CFU of the product should be equivalent to human studies, showing a positive effect. As of now no strict guidelines exist on the dosage of probiotics [121,122]. Consumers should also be aware that for the most part multi-strain probiotics are more effective than monostrain preparations [123]. It is to be assumed that, new probiotic strains with the intent of consumption, should be of human origin (although *Saccharamyces* spp., is an exception), their safety, benefits, and dose ratio, should be clearly stated and assessed (for review see [124]) Before the consumer purchases a probiotic it is recommended to research the probiotic strain best suited for their needs paying particular attention to studies performed in human subjects. Customers should ensure the commercial probiotic dosage is consistent with the dosage used in human studies. An important consideration to keep in mind is that the use of probiotics in sport is still a novel area; therefore, most studies to date are preliminary pilot studies, where the dosage and exact benefit of the strain are not fully optimized, and therefore should be reasoned with caution.

## 6. Probiotics in Treating the Overtraining Syndrome

Overtraining can put the athlete at risk of developing asthma [125], infection such as upper respiratory illness [126,127], gastrointestinal complaints [128] as well as depression and anxiety [129]. Various other symptoms such as immunity suppression and chronic fatigue are also very common [26]. The symptoms can have negative effects for the athlete; impeding progress and decreasing performance. Various studies have explored the positive relationship between athletes and probiotics, and how they can possibly be used to ease or prevent symptoms associated with overtraining. The effects of probiotics on athletes and physically active individuals are summarized in Table 3.

### 6.1. Antioxidant Boosting

Oxidative stress is the imbalance between oxidant and antioxidant levels in the body; it results in the creation of reactive oxygen species (ROS). Our bodies counteract them with special enzymes that neutralize the reactive species and if the species are not neutralized then they can compromise the cell [135]. Diseases such as rheumatoid arthritis, heart disease, Parkinson’s disease and aging can all be linked to oxidative stress. Intense physical regime coupled to the increased oxygen consumptions results in athletes having a higher abundance of reactive oxygen species [130]. Various probiotic species have been reported to have antioxidant activities; *Lactobacillus delbrueckii* ssp. *bulgaricus, Lactobacillus delbrueckii* ssp. *lactis, Lb. acidophilus, Lb. casei* [136]. Studies have also demonstrated the antioxidant activity of *B. animalis*, which was found to absorb hydroxyl radicals and superoxidase anions in vivo, as well as, increasing the general antioxidase activity in murine species [135]. Certain probiotic strains have also been found to manufacture metabolites that have antioxidant abilities such as; *Lb. fermentum* E3 and E18 have been found to produce glutathionine (GSH), and *Clostridium butyricum* has been detected to produce the SCFA butyrate [135]. SCFAs have health-aiding properties and are thought to help with the immune system [137,138] as well as insulin resistance [139]. Martarelli *et al* has investigated if two probiotic strains (*Lb. rhamnosus* IMC 501 and *Lb. paracasei* IMC 502) have any effect on the oxidative stress of athletes during a four-week time frame of intense training [130]. The study came to two findings; firstly, intense exercise significantly increased the oxidative stress in athletes, and secondly, the probiotic course successfully lowered the oxidative stress caused by the intense exercise.

### 6.2. Tight-Junction Protein Modification

**Figure 2:**
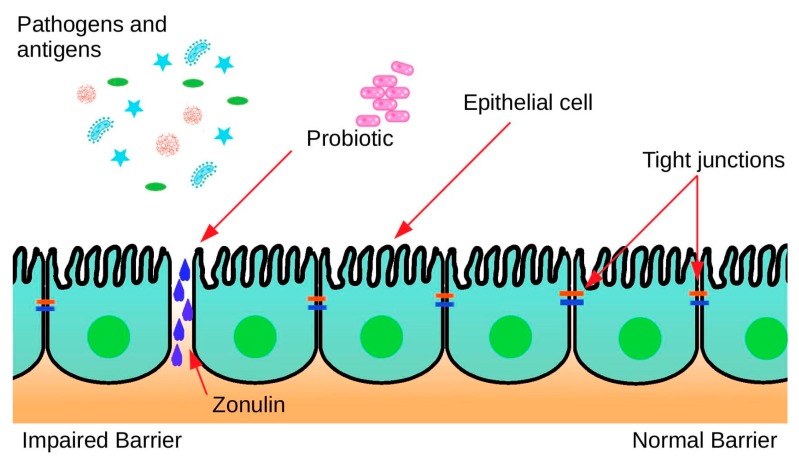
Decreased intestinal permeability.

The mucosal layer of the intestine is made up of several constituents namely; enterocytes and epithelial cells, which are interconnected by tight junctions. This intestinal barrier is pivotal in nutrient and water absorption, while preventing harmful substances entering the bloodstream [140,141]. During intense continuous exercise, cardiovascular and thermoregulatory responses augment the blood flow, increasing the phosphorylation state of the tight-junction proteins consequently increasing their permeability (Figure 2). Increased permeability can cause; vulnerability to allergies and infections, along with various gastrointestinal complaints [142]. A randomized, double-blinded, placebo-controlled trial has investigated two probiotic brands in relation to markers of intestinal barrier integrity on 23 endurance male athletes. The probiotics of choice were Ecologic^®^Performance (*B. bifidum* W23, *B. lactis* W51, *Enterococcus faecium* W54, *Lb. acidophilus* W22, *Lb. brevis* W63, *L. lactis* W58) and OMNi-BiOTiC^®^POWER (*E. faecium* W54, *Lb. acidophilus* W22, *Lb. brevis* W63, *L. lactis* W58, *B. bifidum* W23, *B. lactis* W51). The study investigated the concentration of Zonulin in faeces, a marker of impaired gut barrier. The study concluded that the probiotic treatment effectively decreased Zonulin concentrations in faeces from above normal to below normal, during the 14 days of the study, when compared to a placebo [142].

### 6.3. Immune System Modulation

It has been documented that overtraining can lead to immunosuppression within the individual [26]. The tissue trauma associated with excessive training results in overproduction of cytokines (IL6, TNF-alpha) which subsequently lead to chronic fatigue-like behaviour in athletes, followed by the induction of a humoral response, resulting in the suppression of cell-mediated immunity resulting in a higher chance of infections (for review see [143]).

Much research has focused on potential probiotics and their strain specific effect on the immune system. Throughout the years several different probiotics have been found to influence the host immune system. Studies have shown that *Lb. plantarum* 299v is capable of inducing mucin, MUC2 and MUC3, which is important in hindering pathogen colonization [144,145]. Mucin is a large glycosylated protein which protects the intestines by a minimum of three mechanisms: a) it creates a gel-like structure to trap various molecules; and b) it can bind to various molecules, pathogens or proteins through very specific binding sites and c) through disposal of trapped organisms or proteins [146]. *Escherichia coli* have also been assigned some immunomodulatory functions in recent years, EcN has been found to induce beta-defensins, defensins are small peptides which are the innate immune systems first line of defence, they are produced by epithelial cells of the intestinal tract, skin and lung. Defensins have a broad antagonistic spectrum against various pathogens and are active against both Gram-positive and negative bacteria as well as fungi [147]. A cheese product containing live a culture of *Lb. rhamnosus* HN001 and *Lb. acidophillus* NCFM has been proven to improve the ability of natural killer cells (NK Cells) to kill cancerous cells and phagocytosis in elderly subjects. Clancy et al. [132] investigated if a daily dose of *Lb. acidophilus* could reverse a defect in INF-y secretion in fatigued athletes. The study has concluded that treatment with *Lb. acidophillus* has significantly increased IFN-y secretion in fatigued athletes to comparable levels of the control cohort. Another worthy study has investigated the effect of probiotic supplementation on respiratory infections and gastrointestinal symptoms of 141 marathon runners. The probiotic of choice was *Lb. rhamnosus GG,* compared with a placebo pill. The findings of the study were as follows: there was no significant improvement in the URTI, however, *Lb. rhamnosus* GG decreased the duration of the gastrointestinal symptoms by 33% during the training period, compared to the placebo [148]. Various studies have elucidated immune modulation induced by *Akkermansia*. A study by Derrien et al. determined that *A. muciniphilia* is capable of up regulating genes involved in antigen presentation, as well as aiding in B and T cell maturation [100]. Ottman also investigated *A. muciniphilia* and its immune modulation activity, and concluded that *Akkermansia* could activate Toll like receptor 2 (TLR) and TLR4 and induce IL8 and IL10 [101].

### 6.4. Infection

Overtraining can lower the immune system and in turn result in an “open window” period, where sensitivity to infection is increased [126,149]. Athletes are vulnerable to GI, URTI, and skin infections [150].

To benefit the residential flora, probiotic strains need to show antagonistic activities against pathogens. There are two types of antimicrobials produced by certain probiotic strains: high molecular weight compounds called bacteriocins, and low molecular weight compounds including, but not restricted to: hydrogen peroxide, lactic acid, acetic acid and reuterin [151]. Lactic acid, acetic acid and hydrogen peroxide are the most common antimicrobial compounds secreted by probiotic species. LMW compound production is specific to its species, and the effectiveness of microbial inhibition is directly proportional to the amount of organic acid produced [152]. Bacteriocins are small peptides that have been ribosomally-synthesized which possess antagonistic properties against other bacteria, to which the producing strain is immune (for review see [153]). Bacteriocins can either be narrow spectrum (bactericidal against closely related bacteria) or broad spectrum (bactericidal against a wide variety of strains (for review see [151,154]). They can be classified into three groups, Class 1 bacteriocins are lantibiotics which possess lanthionine or beta-metyllanthionine residues, like nisin, Class 2 can be classified as non-lanthionine containing bacteriocins such as lactacin F, and finally bacteriolysins which are non-bacteriocin lytic proteins like lysostaphin [153]. Bacteriocins are an important trait for a probiotic to possess, they can aid in the survival of the producer within a gut [155], help inhibit the proliferation of other bacteria and function as sensing molecules (for review see [155,156]). The production of bacteriocins is dependent on a variety of factors including, the bacterial species producing the antimicrobial compound, and the environmental conditions present. Different bacteria produce various types of bacteriocins, for example, *Lb. reuteri* is known to produce reuterin, *L. lactis* produces Nisin A, *E. faecalis* DS16 makes Cytolisin, *Lb. plantarum* manufactures Plantaricin S and *Lb. acidophillus* makes acidophilicin. The production of antimicrobial entities is an important probiotic trait. It allows for longer survival of the producer in the intestine, as well as having the potential to outcompete pathogenic bacteria maintaining a healthy bacterial balance in the gut. Athletes tend to be prone to gastroenteritis and other infections, and the antimicrobial compounds produced by the probiotics can help in easing or preventing the symptoms.

### 6.5. Mental Health

The ubiquity of depressive symptoms in elite athletes ranges from 4%–68%, with the consensus that female athletes are more likely to develop depressive episodes than male athletes. Athletes, who take part in individual sports, rather than team-based sports, tend to be more susceptible to depressive symptoms. Elite athletes face risk factors that contribute to depression including, genetic factors, environmental factors, injury, competitive failure, retirement from sport, pain, concussion and of course overtraining [157].

A study by *Sashihara* [133] and colleges investigated if *Lb. gasseri* OLL2809 combined with alpha-lactalbumin decreases symptoms of depression. The study has found that *Lb. gasseri* OLL2809 has successfully elevated symptoms of depression in college athletes. Similarly, Strasser et al. investigated the effect of a multi-species probiotic formulation (*B. bifidum* W23, *B. lactis* W51, *E. faecium* W54, *Lb. acidophilus* W22, *Lb. brevis* W63, *L. lactis* W58) and its possible effect on tryptophan levels, often implicated in the etiology of depression [158]. The study concluded that the multispecies probiotic formulation had a positive effect on the tryptophan-kynurenine pathway by successfully reducing the exercise-induced depletion of tryptophan levels that occur after excessive exercise [134].

## 7. Conclusion and Future Prospects

In conclusion, the relationship between physical fitness and the microbiome is a complex one. Exercise and physical fitness can positively alter the composition of the microbiome and benefit host health, consequently maintaining athletic potential. Studies suggest that different forms of exercise may influence the abundance of different bacterial populations and, hence, alter the microbiome in a different way and, therefore, studies in the future should consider this. As addressed in this review, strenuous exercise can put an athlete in a predicament where they are more prone to infection, inflammation, depression and gut permeability dysfunction. Probiotics offer an effective strategy to prevent or improve those symptoms and allow for continued athletic success without the drawback of the aforementioned symptoms. Future research should aim to clarify the optimal dosage for each individual strain with proven efficacy, as well as establishing whether the probiotic strains colonize our intestine or are simply transient microorganisms with beneficial effects. The impact of probiotics on the athletic population is a new and exciting area, where a limited amount of research has been conducted to date, and although research has shown great promise, little is known about the benefits of probiotics in highly active individuals and ultimately if they benefit from them.

## Figures and Tables

**Table 1:** Studies of exercise and the microbiome.

Subject Group	Microbiome Change	Key Findings	Study Reference
Rugby players	*↑Akkermansia, Prevotella, ↓Ruminococcaceae* *Bacteroides* *Lactobacillus*	*Akkermanisa* was associated with better immunity and gut barrier function while *Prevotella* was correlated to biosynthesis of branched-chain amino acid (BCAA) pathways which help with muscle recovery	[14]
Professional male athletes	*↑Akkermansia* metabolic pathways/higher short-chain fatty acids (SCFA) metabolic pathways	Rugby players had a higher abundance of health-promoting *Akkermansia* genus, which has been associated with an improved gut barrier function and immune function stimulation.	[13]
Cyclists	*↑Prevotella, Methanobrevibacter* *Smithii*	*Prevotella* was correlated to biosynthesis of BCAA pathways which help with muscle recovery, *M. smithii* has been associated with degradation of H_2_ which is used to make ATP/SCFA resulting in a more energetically efficient body	[15]
Sedentary adults challenged to eight week exercise regime	↓in *Archaea* species and an *↑* in microbial diversity	Microbial diversity has been linked to an overall better health	[16]
Marathon runners	*↑Veillonella*	*Veillonella* has been shown to metabolize lactate to SCFA, lower inflammation and increase performance in murine models	[18]
Bodybuilders and Distance runners	*↑Faecalibacterium, Sutterella, Clostridium, Haemophilus, Eisenbergiella* *↓Bifidobacterium, Parasutterella and Eubacterium*	Different sports and their sport specific diets can affect the gut microbiome in different ways	[17]

**Table 3:** Summary of studies using probiotics on physically active and athlete cohorts.

Subject Group	Intervention	Result	Limitations of the Study	References
20 male elite distance runners. Randomized double-blinded, placebo controlled trial,	*Lb. fermentum* VRI-003	↓ risk and severity of respiratory systems ↑ INFƴ	A small sample size	[64]
99 male and female competitive cyclists. Randomized, double-blinded, placebo trial	*Lb. fermentum* (PCC^®^)	↓ severity of GIT symptoms ↓ severity/duration of lower respiratory illness ↓ cytokine imbalance	Inclusion criteria for antibiotics was only four weeks, study relied on self-reported illness, reported a higher rate of lower respiratory illness in females.	[65]
10 male runners. Randomized, double-blinded, placebo trial	*Lb. acidophilus, Lb. rhamnosus, Lb. casei, Lb. plantarum, Lb. fermentum, B. lactis, B. breve, B.bifidum* and *Streptococcus thermophilus*	↑ increased run time to fatigue, small to moderate improvement in gut permeability	Study only investigated males, sample size was too small, short study duration of 4 weeks.	[66]
465 physically active males and females. Randomized double blind placebo controlled trial	*B. animalis* subsp. *lactis* BI-04 (BI-04), *Lb. acidophilus* NCFM and *B.animalis* subsp. *lactis* BI-04 (NCFM and BI-04)	↓ the risk of URTIs by 27%	No separation between recreational and professional athletes, study relied on self-reported illness data, inclusion criteria for antibiotic use was only four weeks.	[72]
30 male elite male rugby players. Randomized, double-blinded, Placebo trial.	*B. bifidum B. longum Lb. gasseri*	↓ in the incidence of URTI/GIT	Relatively small sample size, study only looked at males, short study duration of 4 weeks, relied on self-reported illness data as opposed to measuring immune system markers	[73]
15 resistance-trained men. Randomized, double-blinded, placebo trial.	*B. breve* BR03 and *S. thermophilus* FP4	positive effect on the reduced performance and range of motion followed by intense muscle damaging exercise	Small sample size, looked at males only, didn’t include antibiotic use in inclusion criteria, short study duration of 3 weeks	[74]
24 amateur athletes	*Lb. acidophilus* (CUL60/CUL21), *B. bifidum* (CUL20), *B. animalis* subsp. *Lactis* (CUL34)	↓ incidence and severity of GIT symptoms, both during training and a marathon race	Small sample size, ratio of males to females was skewed, athletic levels weren’t standardized, not double-blinded.	[75]
24 amateur male athletes	*Lb. rhamnosus* IMC 501 and *Lb.* *paracasei* IMC 502	↓antioxidant levels followed by exercise	Small sample size, short study duration of 4 weeks, studied males only, study did not include placebo in control group	[130]
23 endurance male athletes Randomized, double-blind, placebo controlled trial.	EcologicWPerformance or OMNi-BiOTiCWPOWER,	↓ zonulin ↓TNF-alpha	Small sample size, only looked at men, looked at only one marker or impaired intestinal permeability	[131]
27 trained amateur athletes	*Lb. acidophilus* LAFTI^®^	↓fatigue	Small sample size, sample size of control group was significantly lower than test group, the ratio of male to females was skewed, not randomized or double blinded.	[132]
44 university student athletes. Randomized, double-blind, placebo controlled trial.	*Lb. gasseri* OLL2809 combined with alpha-lactalbumin	prevents the exercise induced drops in Natural Killer cells positive effect on minor fatigue ↑mood from a depressive state	Study only looked at males, all participants were at a university level, relied on self-reported illness, short study duration of 4 weeks	[133]
33 highly trained individuals. Randomized, double-blinded, placebo controlled trial.	*B. bifidum* W23, *B.* W51, *Enterococcus faecium* W54, *Lb. acidophilus* W22, *Lb. brevis* W63, and *Lactococcus lactis* W58	↓drops of tryptophan levels caused by intense exercise ↓incidence of URTI’s	Relatively small sample size, women were overrepresented; the severity of illness could not be calculated due to lack of replies from participants.	[134]
